# Dipolar Brush Polymers: A Numerical Study of the Force Exerted onto a Penetrating Colloidal Particle Under an External Field

**DOI:** 10.3390/polym17030366

**Published:** 2025-01-29

**Authors:** A. Fuster-Aparisi, Antonio Cerrato, Josep Batle, Joan Josep Cerdà

**Affiliations:** 1 Departament de Física UIB, Institut d’Aplicacions Computacionals de Codi Comunitari (IAC3), Campus UIB, 07122 Palma de Mallorca, Spainjbv276@uib.es (J.B.); 2 Departamento de Ingeniería de la Construcción y Proyectos de Ingeniería, Escuela Técnica Superior de Ingeniería, Universidad de Sevilla, Camino de los Descubrimientos, 41092 Sevilla, Spain; antoniocerrato@us.es

**Keywords:** dipolar brushes, magnetic brushes, colloids, numerical simulations, Langevin dynamics

## Abstract

Langevin Dynamics numerical simulations have been used to compute the force profiles that dipolar polymer brushes exert onto a penetrating colloidal particle. It has been observed that force profiles are strongly influenced by externally applied fields: at large distances from the grafting surface, a force barrier appears, and at shorter distances a region with lower repulsive forces develops. Furthermore, with the right combination of polymer grafting density, polymer chain length and strength of the external field, it is possible to observe in this intermediate region both the existence of net attractive forces onto the penetrating particle and the emergence of a stationary point. The existence of these regions of low repulsive or net attractive forces inside the dipolar brushes, as well as their dependence on the different parameters of the system can be qualitatively reasoned in terms of a competition between steric repulsion forces and Kelvin forces arising from the dipolar mismatch between different regions of the system. The possibility to tune force profile features such as force barriers and stationary points via an external field paves the way for many potential surface–particle-related applications.

## 1. Introduction

Polymer brushes are soft-matter systems in which polymer chains are anchored or attached to a surface by one of their ends. The advances in chemical synthesis have allowed creating many different types of polymer brushes, and the research on this topic is still very active [1,2,3,4,5,6,7,8,9,10,11,12,13,14]. The properties of brushes and their internal structure have been throughly studied via experiments [13,15,16,17,18,19,20,21,22,23], theory [13,18,24,25,26,27,28,29,30,31,32,33,34,35,36,37,38,38], numerical simulations [27,29,30,31,39,40,41,42,43,44,45], and Self Consistent Field (SCF) approaches [46,47,48,49]. The use of these systems for new applications has also received substantial attention [9,50,51,52,53,54,55,56,57,58,59,60,61,62,63,64,65,66,67,68,69,70].

Another aspect related to the study of polymer brushes is the characterization of the force exerted by a polymer brush onto a penetrating particle. In the case of neutral systems, a full Self Consistent Field Theory (SCFT) suitable for particles of small radii was developed by Kim and Matsen [71]. These authors focused on several important aspects of the brushes, like the free energy penalty, the suitability of the strong-stretching theory of Milner–Witten–Cates, as well as the suitability of the Derjaguin approach. Since then, additional progress has also been achieved by several authors using SCFT frameworks [72,73,74,75]. The brush–particle interaction has also been analyzed in the constraint case of porous substrates by Santo et al. [76].

In addition to SCF approaches, other theoretical frameworks and numerical techniques have been applied to the study of the neutral brush–particle interaction. For instance, Density functional theory (DFT) has been used in predicting polymeric forces for antifouling applications [32]. Monte Carlo [77,78], Molecular Dynamics simulations [79], and Brownian Dynamics [80] have also been used to improve the determination of the brush–particle forces. Other numerical techniques like Dissipative Particle Dynamics have been used in the study of the brush–particle interaction out of equilibrium conditions (shear flow) [81].

Studies of brush–particle interactions have also been carried out in more complex environments like non-spherical penetrating particles [82,83], polymer brushes with mobile grafted chains [74], or systems in which the grafting surface is permeable to the fluid [84]. Without being extensive, in relation to the study of the brush–particle interactions for neutral brushes, we also found studies on the transport of colloidal particles across the polymer brushes [85], on the determination of the depletion forces induced in proteins that are embedded in polymer brushes [86], and on the theoretical development to account for the interaction of polymer brushes with antimicrobial [87], to mention just a few examples.

In addition to neutral polymer brushes, it is also possible to have charged polymer brushes, and the study of the brush–particle interactions in these systems has also received some attention. For instance, the controlled adsorption of nanoparticles on polyelectrolyte brushes via pH has been addressed by Astier et al. [88]. Furthermore, Popova et al. [89] using mean-field Poisson–Boltzmann approximation, have shown that a charged brush uptakes the nanoparticles when the interaction between the brush and the particle promotes changes in the ionization state of the weak cationic and anionic groups on the surface of the nanoparticle.

Another largely interesting type of polymer brush is the dipolar polymer brush in which all or part of the monomers bear an electric or magnetic dipole. These polymer brushes can be made of molecular or chemical polymeric chains, but there also are supramolecular dipolar brushes in which colloidal particles play the same role as monomers in a traditional chemical polymer. In those supramolecular systems, colloidal particles can carry an electric or magnetic dipole. It must be remarked that studies conducted for any one class of dipolar brushes are very relevant to all the other classes: on one hand, magnetic and electric dipolar interactions have the same formal structure except for a constant pre-factor, and on the other hand, all molecular brushes can be mapped onto coarse-grained models.

In the case of molecular electric dipolar polymers, we refer to the seminal work of Stockmayer [90] for a detailed chemical description of them. Studies of the properties of polymer brushes made of electrical dipolar chains [46,91,92] are scarcer than those on neutral and charged brushes. At the molecular scale, magnetic polymeric chains are only found at T < 100 K [93,94], which hampers their use and characterization. At the supramolecular level, the synthesis of magnetic dipolar brushes has received considerable attention. These magnetic systems constitute a particular type of magnetoresponsive thin film surfaces [95,96] for which several synthesis pathways have already been developed [97,98,99,100,101,102,103,104,105,106,107]. Superparamagnetic and paramagnetic brushes have been used to design magnetoresponsive nanoscaled actuators [97] analogous to polymer responsive brushes [108] and micro-metric cilia arrays [109,110,111]. The approach followed to create such brushes mostly consisted of modeling filaments as elastic rods in which the direction and strength of the magnetic dipoles are induced by an oscillating external field. More recently, the study of magnetic brushes made of fully magnetic ferromagnetic filaments has also been addressed [101,112,113,114,115,116,117,118].

The interest in these magnetic or dipolar brushes is driven by their promising potential for technological applications in the areas like actuators and mixers [98,109,119,120], dynamical absorbers [121], micromechanical sensors [122], non-permanent photonic crystals [123], diffraction gratings [124], and medicine [125,126], to mention just a few [127]. Filaments that react to external magnetic fields have several advantages over traditional polymer and polyelectrolyte brushes, which are more constrained in their responsivity to external fields: changes in pH or the electric field have a strong and complex impact not only on the polymer brush but also on most of the soft matter substances. For these reasons, the research of magnetic brushes is evolving into a very promising field.

The characterization of the properties of free magnetic dipolar brushes has been studied in several works [113,114,115] which have been complemented by the work of Mikhailov et al. [46] using a Scheutjens–Fleer self-consistent field (SF-SCF) method. In this collection of works, many observables of these brushes have been studied in detail: monomer density profiles, distribution of free-end segments, brush height, as well as the probability distribution functions of the gyration radii and the end-to-end distance. The structure factors of these brushes and their magnetization have also been computed, and measurements of the internal clustering of the dipoles have been performed, too. Other works have focused on the study of these dipolar brushes in confined slits as well as on their response under flow and in an external applied field [117]. In turn, Cerdà et al. [118] have performed an analysis of how the main brush observables studied in previous works are influenced by the amount of dipolar content in the brush.

To the best of our knowledge, so far no study has focused on the interaction of dipolar brushes with neutral penetrating particles. In this work, we aim to contribute to closing that gap by studying the brush–particle interactions for supramolecular magnetic dipolar brushes in a flat geometry and using spherical penetrating particles with a diameter larger than the separation between adjacent grafted particles, but of the same order of magnitude. Although we exemplify our study via magnetic colloidal systems, all results derived in this work can be widely extended to all classes of dipolar systems (molecular and supramolecular classes).

In particular, we will study the dependence of the force profiles for these systems on several parameters: the radius of the penetrating particle, the fraction of dipolar particles present in the brush, the strength of the externally imposed field, and the grafting density of the brush. It will be shown that for some range of brush–particle parameters and field strengths there is an attractive zone for the colloidal particle inside the brush, as well as other interesting features that show that dipolar brushes may exhibit a behavior much richer than the behavior of neutral brushes. One of the main aims of this study is to contribute to pinpointing the regions within the space of parameters that can be of high interest in forthcoming studies about dipolar polymer brushes.

The present manuscript is organized in the following way. In the next section, the numerical model and the details of the simulations are described. In this section, we also provide examples that show the correspondence between our model for dipolar brushes and the different classes of dipolar brush systems. The main findings are provided in Section 3. Finally, the summary of our main results is provided in Section 4.

## 2. Numerical Method

**Figure 1:**
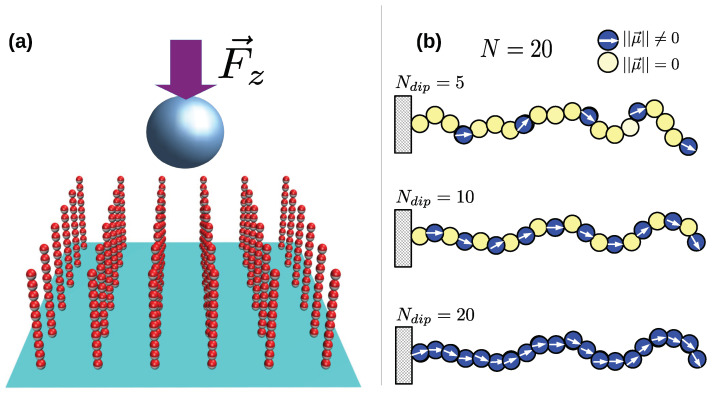
(**a**) depicts a schematic view of the system under study, (**b**) shows some of the chain dipolar sequences tested in this work, $N_{dip}$ and *N* indicate the number of dipolar monomers and the total number of monomers per chain, respectively.

The dipolar brushes are modeled as a regular squared lattice array of $N_{c}\times N_{c}$ spring-bead chains made of *N* monomers each, and all of them are grafted by one of their ends at the plane x-y at z=0. The brush is placed into a cubic box of edge $L_{e}$ where periodic boundary conditions along x-y directions are allowed, and a large non-dipolar colloidal particle of radius $R_{e}$ is placed with its center at position $\left(L_{e}/2,L_{e}/2,{z_{e}}_{o}\right)$, where ${z_{e}}_{o}$ is larger than the length of a fully extended chain. See Figure 1a. In our simulations, several types of chain sequences are tested in order to understand how the brush structure depends on the number of dipolar monomers present in the chains. In any given simulation, all chains have the $N_{dip}$ dipolar monomers equispaced along the chain sequence where the free-end monomer always carries a dipole. See a scheme in Figure 1b. All chain beads, dipolar and non-dipolar, are assumed to be spheres of diameter $\sigma_{e}$ and mass $m_{e}$.

Subindex *e* has been used to denote the experimental values of the physical quantities we use. Note that in our work, reduced units are used instead. For instance, all length scales in the simulations are measured in units of the bead diameter, and therefore, the corresponding reduced value of any experimental length $l_{e}$ is $l=l_{e}/\sigma_{e}$. In this way $R=R_{e}/\sigma_{e}$, $L=L_{e}/\sigma_{e}$, $z_{o}={z_{e}}_{o}/\sigma_{e}$ and due to the previous definition, the diameter of all chain beads or monomers is set to σ=1.

In order to link the monomers that form a chain, we use a finite extension non-linear elastic (FENE) potential [128] among adjacent monomers in the chain sequence,

(1) $$U_{s}\left(r\right)=-\frac{1}{2}K_{s}\Delta r_{max}^{2}\ln\left[1-{\left(\frac{r-r_{0}}{\Delta r_{max}}\right)}^{2}\right],$$

where the constants of the potential are set to $r_{0}=\sigma$, Δr=1.5σ and $K_{s}=15$.

All short-range isotropic interactions between monomers in the present model are of the Lennard–Jones (LJ) type. Thus, the monomers interact among themselves according to the potential

(2) $$U_{att}\left(r\right)=V_{tsLJ}\left(r,\sigma ,1,r_{cut}=2^{1/6}\;\sigma \right)+V_{tsLJ}\left(r,\sigma ,\varepsilon ,r_{cut}=2.5\;\sigma \right)$$

where *r* is the distance between the centers of the monomers *i* and *j*, i.e., $r=|r_{i}-r_{j}|$, and $V_{tsLJ}$ is a truncated-shifted Lennard–Jones potential [129],

(3) $$V_{tsLJ}\left(r,d,U_{o},r_{c}ut\right)=\left\{\begin{array}{cc} U_{LJ}\left(r\right)-U_{LJ}\left(r_{\mathrm{cut}}\right), & \mathrm{for}r<r_{\mathrm{cut}}\\ 0, & \mathrm{for}r\geq r_{\mathrm{cut}} \end{array}\right.,$$

where $U_{LJ}\left(r\right)=4U_{o}\left[{\left(d/r\right)}^{12}-{\left(d/r\right)}^{6}\right]$. The first truncated-shifted LJ (cut-off $r_{cut}=2^{1/6}\sigma$) in Equation (2) stands for the core repulsive part and the second truncated-shifted LJ ($r_{cut}=2.5\sigma$) for the attractive part. The reason to model the interaction of the short-range part of the beads with two truncated-shifted potentials and not just a simple LJ is to always have roughly the same repulsive core interaction independent of the value of attractive depth well ε. This allows for a much easier comparison between different values of ε and the effective particle size is kept roughly similar in all cases.

In the simulations, all the energy values are given in units of the experimental well depth $\varepsilon_{e}$ between two monomers. Thus, the expression to map a physical energy value $U_{e}$ to its counterpart in the reduced unit system *U* is $U=U_{e}/\varepsilon_{e}$. In the same way, the Boltzmann constant is chosen to be k=1 in reduced units, and therefore, the reduced temperature is $T=k_{e}T_{e}/\varepsilon_{e}$.

The interaction between any monomer and the penetrating colloidal particle of radius *R* is modeled via a purely repulsive potential

(4) $$U_{rep}\left(r\right)=V_{tsLJ}\left(r,R+\sigma /2,1,r_{cut}=2^{1/6}\;\left(R+\sigma /2\right)\right)$$

In addition, all the chain monomers present in the system exhibit a steric repulsion with the grafting surface located at the x–y plane that is modeled using a standard 9–3 WCA (Weeks–Chandler–Andersen) potential:

(5) $$V_{wall}=\left\{\begin{array}{cc} U_{wall}\left(r\right)-U_{wall}\left(r_{\mathrm{cut}}\right), & \mathrm{for}r<r_{\mathrm{cut}}\\ 0, & \mathrm{for}r\geq r_{\mathrm{cut}} \end{array}\right.,$$

where $U_{wall}\left(r\right)=\left[{\left(\sigma /r\right)}^{9}-{\left(\sigma /r\right)}^{3}\right]$ and $r_{cut}=3^{1/6}\sigma$ in this case. The grafted monomers of the chains are placed at $z=3^{1/6}\sigma$, which corresponds to $V_{wall}=0$. No repulsive interaction is set between the grafting surface and the penetrating particle since the penetrating particle is never in close contact with the surface, and we want to focus specifically on the influence of the monomers of the brush on that particle.

A point dipole $\mu_{e}$ is assigned to each dipolar monomer. The dipoles are located at the center of the monomers, and fixed within the particle body so that its rotation is coupled to the rotation of the monomer. In what follows, we will assume Gaussian electromagnetic units in all our formulas where dipolar moments or external fields appear. The modulus of the dipole moments in reduced units using Gaussian electromagnetic units is $\mu^{2}=\mu_{e}^{2}/\left(\sigma_{e}^{3}\varepsilon_{e}\right)$, where |μ|=μ. There are two types of interactions related to the dipoles. The first one is the dipole–dipole interaction:

(6) $$U_{\mathrm{dip}}\left(r_{ij},\mu_{i},\mu_{j}\right)=\frac{\mu_{i}\cdot \mu_{j}}{r^{3}}-\frac{3\left[\mu_{i}\cdot r_{ij}\right]\left[\mu_{j}\cdot r_{ij}\right]}{r^{5}},$$

where $r_{ij}=r_{i}-r_{j}$ is the displacement vector between particles *i* and *j*. In the case of magnetic colloidal polymer brushes, reasonable values of μ=|μ| depend, in general, on the composition and size of the nanoparticles, but in usual ferrofluids it typically does not exceed $\mu_{e}^{2}/\left(kT_{e}\sigma_{e}^{3}\right)$∼10. The long-range dipole–dipole interactions in this half-space geometry are calculated using a twofold step. First, dipolar interactions are calculated using the standard $P^{3}M$ algorithm $\left(dP^{3}M\right)$ [130] that implicitly assumes periodic boundary conditions along all directions; second, a dipolar layer correction (DLC) [131] is applied which discounts the effect of the excess of infinite replicas added along the *z* direction in the first step of the calculation. The combined use of $dP^{3}M$ and DLC methods allows for a much faster calculation of the dipolar long-range interactions than with the traditional two-dimensional dipolar Ewald summations [132] adapted to slit geometries; computer times scale with the total number of dipolar particles $N_{d}=N_{c}^{2}\;N_{dip}$ present in the system as $N_{d}\ln\left(N_{d}\right)$ and $N_{d}^{3/2}$, respectively. The level of accuracy of the algorithm for computing dipolar forces and torques is set to δ∼10^−4^ in our simulations.

The dipoles are also allowed to interact with an external field $H_{e}$. Its orientation is kept fixed along the z-direction, so that in reduced units H=(0,0,H), where $H=H_{e}\sqrt{\sigma_{e}^{3}/\left(\varepsilon_{e}\right)}$. Therefore, the Zeeman energy can be written as follows:

(7) $$U_{H}\left(H,\left\{\mu_{i}\right\}\right)=-\sum_{i=0}^{N}H\cdot \mu_{i}=-H\sum_{i=0}^{N}\mu_{z_{i}}.$$

The numerical simulations are performed using Langevin dynamics. In this approach, all the monomers (except the grafted monomers and the penetrating particle whose positions are not updated at each time step) are moved according to the translational and rotational Langevin equations of motion that, for a given particle *i*, are as follows [133]:

(8) $$\begin{array}{c} M_{i}\frac{dv_{i}}{dt}=F_{i}-\Gamma_{T}v_{i}+\xi_{i}^{T}+\Gamma_{T}\dot{\gamma }z_{i}\hat{x} \end{array}$$

(9) $$\begin{array}{c} I_{i}\cdot \frac{d\omega_{i}}{dt}=\tau_{i}-\Gamma_{R}\omega_{i}+\xi_{i}^{R} \end{array}$$

where $F_{i}$, and $\tau_{i}$ are the total force and torque acting on the particle *i*, respectively. $\Gamma_{T}$ and $\Gamma_{R}$ are the translational and rotational friction constants. The dipolar component of the torque, $\tau_{i}^{\left(dip\right)}$, can be computed as

(10) $$\tau_{i}^{\left(dip\right)}=-\mu_{i}\times \nabla_{\mu_{i}}\left(U_{\mathrm{dip}}+U_{H}\right).$$

$M_{i}$ and $I_{i}$ are the mass and the inertia tensor of the colloid, and $\xi_{i}^{T}$ and $\xi_{i}^{R}$ are Gaussian random forces and torques, each of zero mean value and satisfying the usual fluctuation-dissipation relations

(11) $$\begin{array}{ccc} \langle \xi_{i\alpha }^{T}\left(t\right)\xi_{j\beta }^{T}\left(t^{\prime }\right)\rangle & = & 2kT\Gamma_{T}\delta_{ij}\delta_{\alpha \beta }\delta \left(t-t^{\prime }\right), \end{array}$$

(12) $$\begin{array}{ccc} \langle \xi_{i\alpha }^{R}\left(t\right)\xi_{j\beta }^{R}\left(t^{\prime }\right)\rangle & = & 2kT\Gamma_{R}\delta_{ij}\delta_{\alpha \beta }\delta \left(t-t^{\prime }\right), \end{array}$$

where α and β denote x,y,z for translation and rotation in Cartesian coordinates. Equations of motion (8) and (9) are a reasonable approach when the size particles is such that sedimentation forces are negligible. In the simulations, $t=t_{e}\sqrt{\varepsilon_{e}/\left(m_{e}\sigma_{e}^{2}\right)}$, where $m_{e}$ is the real mass of the monomers; $F=F_{e}\sigma_{e}/\varepsilon_{e}$, and $\tau =\tau_{e}/\varepsilon_{e}$. For equilibrium simulations, the values of the mass, the inertia tensor, as well as friction constants $\Gamma_{T}$, and $\Gamma_{R}$ are irrelevant because the same equilibrium state is reached independently of their value. Only the dynamics to attain such an equilibrium state may show differences. For the sake of simplicity, the particle mass of the monomers is chosen to be m=1 and the inertia tensor is chosen to be the identity matrix in order to ensure isotropic rotations I=1. $\Gamma_{T}=1$ and $\Gamma_{R}=3/4$ have also been chosen because these values have been observed to produce a conveniently fast relaxation to the stationary. The reduced time step is set to δt=0.0005 in order to ensure a correct integration of the equations of motion.

To make sure that the results do not depend on the initial conditions, and to improve statistics, three independent runs have been carried out for each set of brush–particle parameters. All simulations have been performed using the package ESPResSo [134] version 3.3.1.

### 2.1. Brush Setup and Initial Equilibration Protocol

In this work, we focus on the study of polymer brushes having their grafted chains arranged in a square lattice. In the first stage, the grafted monomers of each chain are fixed following a squared lattice pattern in the x-y plane. The penetrating particle is then placed at $\left(L/2,L/2,z_{o}\right)$ with $z_{o}$ being larger than the full extended length of the polymer chains that the brush will contain. This way, the initial setup of the chains and the initial equilibration of the brush will not be perturbed by the presence of the penetrating particle.

In the second stage, polymer chains are grown by sequentially placing the other monomers that form the chains following a directed self-avoiding random walk (d-SAW) with z as the preferential growth direction. Although growing the chains following a pure self-avoiding random walk (SAW) is also a possibility, our experience from previous works has shown that in the case of brush dipoles, a d-SAW is slightly more efficient in terms of computational cost to reach thermal equilibration, especially when an external field will be applied to the brush.

Subsequently, the polymer brush is warmed up at T=1 for $2\times 10^{5}$ integrations with the dipolar interaction turned off while the time step is slowly increased from $10^{-3}\;\delta t$ to 0.05δt. Right after, dipolar interactions and the external field are turned on. If the simulation is performed at T=1 a second warm-up stage, consisting of $5\times 10^{5}$ integrations, is performed while gradually raising the time step from $10^{-1}\;\delta t$ to δt. For simulations at T<1, an annealing process using a gradual time step increase from time step $10^{-1}\;\delta t$ to δt is performed: the temperature is varied from T=1 to its final value by performing a set of five annealing cycles of $10^{5}$ steps each.

Once the warming stages have concluded, the initial thermal equilibration of the brush is performed maintaining the penetrating particle fixed during the whole process at position $\left(L/2,L/2,z_{o}\right)$. The brush is equilibrated for a period of $10^{6}\;e^{1/T}\;\delta t$. T=0.5 in all our simulations; therefore, the equilibration is performed over $7.38\times 10^{6}$ time steps. We ensure that the equilibration is correct by monitoring several observables of the system, like the total energy, the end-to-end and gyration radius distributions of the polymer chains, and the bond-length distribution.

### 2.2. Brush–Particle Force Measuring Protocol

Once the brush has been initially equilibrated, we start the descending sequence of the penetrating particle into the brush. The force exerted by the brush onto the penetrating particle will be measured at a discrete set of decreasing distances between the grafting surface and the center of the penetrating particle: ${\left\{z_{i}\right\}}_{i=1}^{i=N}$.

The descending from $z_{o}$ to $z_{1}$ and, in general, from any $z_{i}$ towards $z_{i+1}$ is always performed as follows: an attempt to move the penetrating particle one hundredth of the distance $\Delta z=z_{i}-z_{i+1}$ is executed. If no overlap of the penetrating particle with the monomers is observed, then the displacement is realized and another displacement of Δz/100 is attempted. Otherwise, a further integration of $100\;e^{1/T}\delta t=738$ steps is performed, before attempting a new displacement of the penetrating particle. This iterative process is performed until the penetrating particle reaches the new position of measurement $z_{i+1}$. When the penetrating particle reaches a z-position of measurement, the coordinates of the penetrating particles are fixed again, and the brush is further reequilibrated for another period of $10^{6}\;e^{1/T}\;\delta t$ which in our case amounts to $7.38\times 10^{6}$ time steps. This lengthy re-equilibration is performed to ensure that the brush, perturbed by the penetrating particle, has reached again the thermal equilibrium for this new setup.

Once reequlibration at a given $z_{i}$ is conducted, the force-measuring stage starts. We create an averaged measure of the force exerted by the monomers of the polymers onto the penetrating particle by sampling the force at intervals of $100\;e^{1/T}\delta t=738$ time steps for a total period of $2\times 10^{6}\;e^{1/T}\;\delta t=14.75\times 10^{6}$ time steps. The sampling interval is chosen to ensure that measures are largely uncorrelated while maintaining the computational cost of the simulations affordable. Supplementary Material Figure S1, shows sampled forces for z=8,12,16 that are provided for a particle–brush system with parameters similar to those from Figure 3b.

Once the measurement state for $z_{i}$ is concluded, the descending stage of the penetrating particle resumes until the next $z_{i+1}$ is reached. The simulation ends once the penetrating particle reaches the final surface–particle distance $z_{N}$ and the brush–particle forces at that distance are measured.

### 2.3. Correspondence Between the Numerical Model and Experimental Dipolar Brushes

In order to facilitate the comparison with experiments and other works in which dipolar brushes are studied, it is convenient to define a set of non-dimensional parameters. The first one is the strength ratio of the short-ranged attractive interaction to the dipolar interaction

(13) $$\eta \equiv \frac{\varepsilon \sigma^{3}}{\mu^{2}},$$

where the two monomers are considered to be in close contact, $r_{ij}=\sigma$, and with their dipoles oriented in a nose–tail conformation. Two dipoles *i* and *j* are said to be in a nose–tail conformation when $\mu_{i}\cdot \mu_{j}=\mu^{2}$, and $\mu_{i}\cdot r_{ij}=\mu_{j}\cdot r_{ij}=\pm \mu \;r_{ij}$. The nose–tail alignment is the conformation that minimizes the dipolar energy in Equation (6). In this study, we focus on two different values, η=0, that corresponds to a dipolar brush in a good solvent and η=0.05 for a brush in a bad solvent.

A second parameter of widespread use is the dipolar coupling parameter,

(14) $$\lambda \equiv \frac{\mu^{2}}{kT\sigma^{3}},$$

which measures the relative strength of the dipolar interaction (same conditions as in the definition of η) in relation to the thermal energy. In this work, we set λ=10. A third parameter of interest when an external field is applied is the Langevin parameter,

(15) $$\alpha \equiv \frac{\mu \cdot H}{kT},$$

which compares the maximum energy per dipole associated with the external field and the thermal energy. Several field strengths have been sampled in the range α∈[0,45]. In what follows, we will only consider fields oriented perpendicular to the grafting surface of the brush and pointing away from the surface (along the positive z-axis in our system). Finally, another important parameter to characterize the system is the chain grafting density

(16) $$\sigma_{g}\equiv \frac{N_{g}}{L^{2}}$$

where $N_{g}$ is the number of grafted chains in a square of size *L*. The sampled values for the grafting density are $\sigma_{g}=\;0.04,\;0.0625,\;0.0765,\;0.09$. It should be noted that these grafting densities do not cover the mushroom regime which could be of interest in the study of penetrating particles of a much larger diameter. In this work, we focus on the case where the diameter of the penetrating particle is larger but of the same order of magnitude than the distance between adjacent grafted chains.

As mentioned in the introduction section, there are several classes of dipolar brushes. In the case of supramolecular magnetic dipolar brushes, reasonable values of μ=|μ| generally depend on the composition and size of the colloidal particles. For reference, colloidal particles found in common commercial ferrofluids usually do not exceed values (in reduced units) of μ∼10, except in the case of cobalt nanoparticles, which are substantially higher. In order to conduct an estimation of what can be a reasonable range for the parameter $\varepsilon_{e}$ in these systems, we propose two different approaches. The first approach is to consider the interaction potential predicted by the DVLO theory: many systems of ferrofluids are stabilized against flocculation using charges on their surfaces. For instance, if we assume for instance [135] a particle of radius a=133 nm and a Hamaker constant of $A=3.7\times 10^{-21}$ J, and $\psi_{o}=0.01$ V in a water solvent, the result leads to an interaction energy between two colloidal particles close to $2.5\;k_{e}T_{e}$ for a distance between surfaces of 5 nm. The interaction energy decreases to approximately $0.25\;k_{e}T_{e}$ when the separation between surfaces is 10 nm. A second approach is to consider colloidal particles that are stabilized through small layers of some short-chain molecule, for example, oleic acid. In this case, we can conduct an estimate of $\varepsilon_{e}$ using the Rosensweig potential for sterically stabilized magnetic particles [136]. If we choose a Hamaker constant $A=10^{-19}$ J, a surface density of stabilizing polymer of ξ=1 nm^−2^, a thickness of the stabilizing polymers of δ=2 nm, and a core particle diameter of 10 nm, the Roensweig potential predicts a peak barrier of $25\;k_{e}T_{e}$ at a surface-to-surface distance of approximately 0.8 nm for this case. Therefore, taking into account the values obtained from both approaches, it seems reasonable to estimate that in these supramolecular systems $\varepsilon_{e}/\left(k_{e}T_{e}\right)\in \left[2.5,\;25\right]$. This range for ε leads to values of the non-dimensional parameter η∈[0.1,1.0] when μ∼ 5 (in reduced units). The relation between $H_{e}$ and the Langevin parameter α can be obtained using expressions from Section 2 together with Equation (15). If we want to express the values of the field in the International System of Units rather than Gaussian units, we should observe the relation $\mu^{\left(Int\right)}=\sqrt{4\pi /\mu_{0}}\;\mu^{\left(Gauss\right)}$, where $\mu_{0}$ is the vacuum magnetic permeability. Thus, if we assume $\varepsilon_{e}/\left(k_{e}T_{e}\right)\in \left[2.5,\;25\right]$, and we assume we have dipoles of module μ=5 (in reduced units), then a value of α=10 corresponds to a strength of the external field in the range α∈[0.008,0.026] Tesla.

Performing a similar estimation for the experimental parameters in the case of molecular dipolar brushes is more involved: our simulations correspond to a coarse-grain description of the chemical polymer chains. Therefore, in this case, the so-called monomers in our simulations cannot be considered as true chemical monomers but as blobs containing a large number of those chemical monomers, as well as counterions and salt ions if that is the case. Furthermore, dipolar groups can be firmly attached either in parallel or perpendicular to the backbone of the polymer, or be located in a flexible side chain. In addition, the dielectric permittivity filled by the monomers may strongly differ from that of the bulk solvent in which they are immersed. For polar polymers in which the polarization arises from polar covalent bonds, typical values for the electrical dipole associated with a chemical monomer usually range between 0.1 and 2 Debye [90,137], although in some cases it can reach up to 9 Debye [137,138]. In the case of zwitterions, the electrical dipoles are substantially higher: from 11.9 to 15.7 Debye for hydrated glycine [139,140], and in some molecules and peptides the dipole moments can reach values of 20–150 Debye [141,142]. In the case of polyzwitterions the moieties can have also large values for their electrical dipole moment. For instance in the case of poly(2-methacryloyloxyethyl phosphorylcholine) (pMPC), the dipole moment of each moiety is 18.7 Debye [143].

In order to provide a reasonable estimation of the parameters in the case of molecular dipolar brushes, we will assume a brush made of polyzwitterion chains containing $5\times 10^{3}$ chemical moieties, with each one of them having a segment length b=0.5 nm and carrying a dipole of 20 Debye. As we perform a coarse-graining in which the set of all chemical moieties is usually split into 20 large blobs (our monomers in the simulation), we find that we have g=250 moieties per blob. Since the steric interaction at close contact between these blobs in our simulation has an energy of interaction of the order of kT, we can assume that $\varepsilon_{e}$∼$k_{e}T_{e}$. This fact implies that our blobs roughly coincide with the concept of de Gennes’ thermal blobs. Therefore, the size of such blobs can be estimated using de Gennes’ theory to be $\xi_{T}=b\;\sqrt{g}$∼8 nm. Now, we make the assumption that because our blobs are thermal blobs, the chemical monomers inside them are expected to exhibit a behavior close to an ideal chain. For this reason, the net dipolar moment of the blob must be expected to be only a fraction of the theoretical g×20 Debye that could be the maximum value if all chemical moieties had their dipoles perfectly aligned in the same direction. In principle, the exact net dipole associated with a blob will depend on many fine details of the chemical polymers. Nonetheless, we can attempt to conduct a rough approximation to obtain an estimate of the experimental parameters. We will assume that the chain enters the spherical blob and exits it from the opposite side and that dipoles are parallel to the backbone of the chain. In such circumstances, the addition of the dipolar moment of the *g* dipoles should be roughly equal to the sum of the dipolar moments formed by a straight path between both points formed by $\xi_{T}/b=16$ moieties.

Under such assumptions, we obtain that the net dipole momentum of the blob is, therefore, 16×20=320 Debye, which is about 6.4% of the maximum dipolar moment that could exhibit the blob. Now that we have a rough estimation of the size of the blobs and the net dipole they carry, we can evaluate the value of the parameter λ. We should recall that in this manuscript we make use of Gaussian units, and to convert between the International System of Units and Gaussian units we need to take into account the expression $\mu^{\left(int\right)}=\sqrt{4\pi \epsilon_{o}}\;\mu^{\left(Gauss\right)}$. Thus, if we assume that two blobs carry a dipole of 320 Debye at their centers, and that they are in close contact (the distance between their centers being $\xi_{T}$), then we obtain a value of λ=4.94. For such a polymer brush, the Langevin parameter with a value of α=10 corresponds with an external electrical field with a strength of E=37 MV/m. In case we assume λ=10, as we have in our simulations, the blob should contain a net dipole moment equivalent to having approximately 8% of their moieties aligned. In that case, the Langevin parameter of α=10 will correspond to an electrical field E=29 MV/m. As a reference, distilled water exhibits dielectric breakdown around 65–70 MV/m and polyethylene is in a range of 19–160 MV/m. It is also known that dielectric films tend to exhibit larger values of their threshold of dielectric breakdown than bulk samples made of the same material.

## 3. Results and Discussion

In what follows we will focus on the study of how the force created by the monomers of the dipolar brush on the penetrating particle depends on the distance between the grafting surface of the brush and the center of that particle. For the sake of simplicity, hereafter, we will refer to the set of those forces as the force profile of the brush $F_{z}\left(z\right)$, and the aforementioned distance as the surface–particle distance *z*.

**Figure 2:**
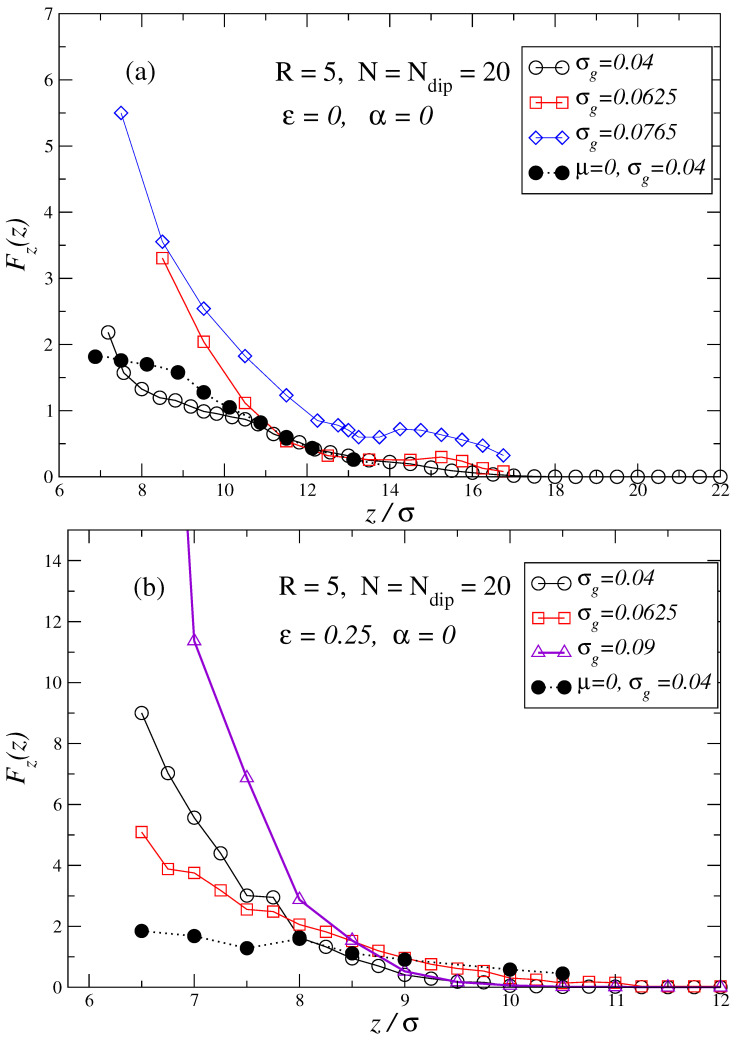
Force profiles along the direction perpendicular to the grafting surface of the dipolar brush as a function of the distance *z* between the grafting surface and the penetrating particle of radius R=5. The chains of the brushes are fully dipolar $N=N_{dip}=20$, and no external field is applied α=0. Several brush grafting densities are shown in each plot: $\sigma_{g}=\;0.04,\;0.0625,\;0.0765,\;0.9$. Plot (**a**) depicts the force profiles for brushes in good solvent ε=0, while plot (**b**) depicts the force profiles for brushes in a bad solvent ε=0.25 (sticky chains condition). Filled black symbols in each plot represent the force profile obtained for the non-dipolar brush case, μ=0, at grafting density $\sigma_{g}=0.04$.

The results obtained from our simulations for the force profiles of dipolar brushes at zero field can be summarized in Figure 2a,b. This figure corresponds to a penetrating particle of diameter R=5 and several grafting densities $\sigma_{g}$. In this figure, the brushes are fully dipolar and the chains have twenty monomers each, with their dipole located at the center of the monomer ($N=N_{dip}=20$). For the sake of comparison, we have also added the force profiles for non-dipolar brushes for $\sigma_{g}=0.04$. For brushes in good solvent, as seen in Figure 2a, the differences between non-dipolar and dipolar brushes for the set of tested parameters show very slight differences except at short surface–particle distances. Conversely, as Figure 2b shows, non-dipolar brushes in bad solvents exhibit a larger range of action and force profiles with a less abrupt increase in the repulsive force when compared with their dipolar counterpart. It is known from previous works [113], that in dipolar brushes, especially in a bad-solvent case, the entanglements induced by dipolar interactions inside the brush create a more compact brush structure than in the case of non-dipolar brushes. This explains the observed shorter action range for the dipolar brushes, and why when the dipolar brush starts to be quite compressed by the penetrating particle, repulsive forces are higher.

**Figure 3:**
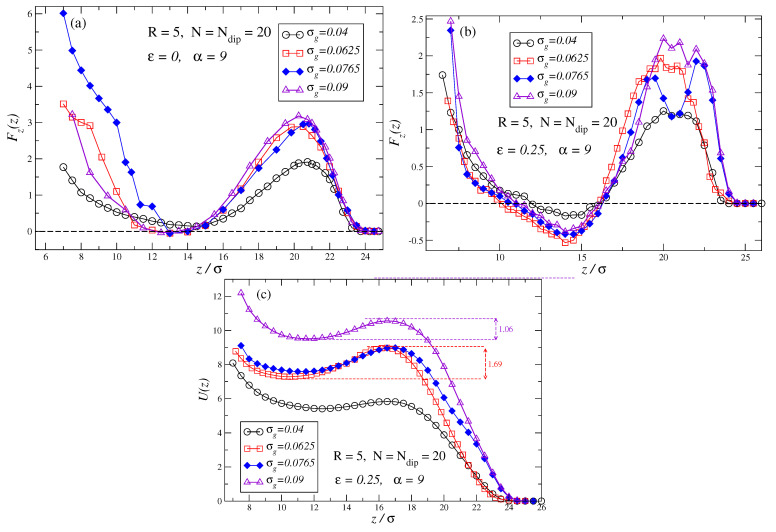
(**a**,**b**) shows force profiles for same brushes as in Figure 2 but now under and external moderate field α=9. Plot (**a**) depicts the force profiles for brushes in good solvent ε=0, while plot (**b**) depicts the force profiles for brushes in a bad solvent ε=0.25 (sticky chains condition). (**c**) shows the potentials obtained from the integration of the forces shown in (**b**), as in our simulations T=0.5, $2U=U_{e}/\left(k_{e}T_{e}\right)$ (see Section 2).

**Figure 4:**
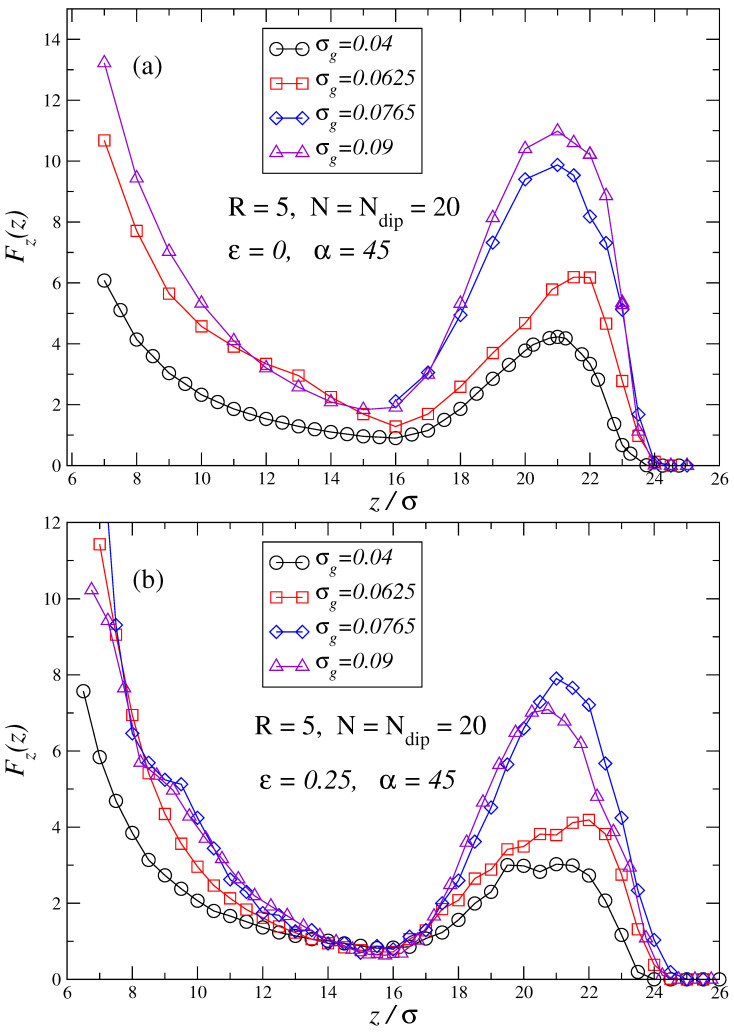
Force profiles for same brushes as in Figure 2 and Figure 3 but now at high field α=45. Plot (**a**) depicts the force profiles for brushes in good solvent ε=0, while plot (**b**) depicts the force profiles for brushes in a bad solvent ε=0.25 (sticky chains condition).

Previous results at zero field are not very surprising. They can be inferred by linking the monomer density profiles, studied in earlier works [113], to the steric hindrance they produce on the penetrating particle. Nonetheless, force profiles suffer a very important transformation when an external field is applied to the dipolar brushes. This can be observed in Figure 3a,b for α=9 and Figure 4a,b for α=45. In those plots, all remaining system parameters are kept equal to those in Figure 2a,b. Results show the appearance of an initial steep barrier that hampers the penetration of the particle. This barrier is followed by an intermediate region where the repulsive force decreases before increasing again at the very short surface–particle distances. In fact, if the grafting density of the brush is large enough, there exists a region in which the brush exerts an attractive force on the penetrating particles (negative values for the force on the force profile). The existence of such attractive forces between the brush and the penetrating particle leads to the existence of a stationary point. The development of a stationary point has important consequences for practical applications as it allows for the intake and retention of colloidal particles or large molecules inside the brush when the field is active, and its ulterior release when the external field is removed.

A comparison of Figure 3a for good solvent (ε=0), and Figure 3b for bad solvent (ε=0.25) shows that in a bad solvent, the attractive forces become stronger and the range of surface–particle distances over which the brush exerts an attractive force is much broader. In addition, the comparison reveals that for brushes in bad solvents, the initial force barrier at large distances is slightly weaker. In contrast to the zero field case, now profiles for both bad and good solvents exhibit quite similar ranges of action. This can be understood in terms of the fact that in both solvent conditions, the chains are quite stretched towards the bulk due to the aligning effect of the external field which dominates and leads to very similar chain persistence lengths in both cases.

Figure 3b shows that the largest attractive force (the most negative) is obtained for a grafting density of $\sigma_{g}=0.0625$. Therefore, one must conclude that given a field strength and a value for the attractive interaction among monomers, ε, there is an optimal value of the grafting density that maximizes the attraction that the brush exerts on the penetrating particle. It should be also noted that for a given dipolar brush, by tuning the strength of the external field, one can tune the properties of the force profiles and obtain the strongest possible attraction between the penetrating particle and the brush.

Figure 3c shows the potentials associated with the forces in Figure 3b. We have taken the case of an unperturbed brush with the particle fully outside as the reference state. Since in our simulations T=0.5, and by the definitions of $U=U_{e}/\varepsilon_{e}$ and $T=k_{e}T_{e}/\varepsilon_{e}$ (see Section 2), then $2U=U_{e}/\left(k_{e}T_{e}\right)$. Therefore, when the particle attempts to penetrate the brush, it must confront a potential barrier of several kT’s. In the intermediate range of surface–particle distances, the penetrating particle enters a well potential whose depth is of the order of magnitude of $2k_{e}T_{e}$ (for the particle–brush parameters and external field applied in Figure 3b). It should be remarked that these potential barriers and wells can be easily activated/deactivated at will by just turning on/off the external applied field.

Figure 4a,b, shows the force profiles for similar brush parameters as in previous figures but for α=45. By comparing these new force profiles with the corresponding ones in Figure 3a,b, it can be observed that two effects take place when the strength of the external field is substantially increased: the amplitude of the initial force barrier is increased, and attractive forces cease to exist as the force profile becomes fully repulsive. Nonetheless, even for such high fields, an intermediate region with substantially lower repulsive forces still exists. It should be observed that the range of action of the force profiles for α=45 remains approximately the same as for α=9, which implies that for α=9 brush chains are already in a quite extended conformation. Consequently, a further increase in the external field strength does not substantially modify the extension of the chains although it has deep effects on the force profiles of the brushes.

It remains to be explained why there is such a dramatic change in the force felt by the penetrating particle when an external field is applied. The initial force barrier felt by the penetrating particle is clearly caused by the steric interactions among the monomers of the chains and the penetrating particle: the enhanced persistence length of the dipolar chains in an applied external field displaces a large amount of monomers far from the grafting surface, and brushes become more resistant to compression. The drop in the repulsive forces once the particle has overcome the initial force barrier can be understood in terms of Kelvin forces: these forces arise from the dipolar mismatch between the non-dipolar parts of our system (the grafting surface and the penetrating particle), and the dipolar media created by the dipolar monomers of the brush chains. For instance, a clear example of such Kelvin forces in soft matter occurs in the magnetophoresis of spheres of weak magnetic materials, where the sum of all these forces onto a single particle immersed in a medium can be summarized in the following expression [144]:

(17) $$F_{mag}=\frac{\chi_{p}-\chi_{m}}{2\mu_{o}}V\nabla {\left(B\right)}^{2},$$

where, $\chi_{p}$ and $\chi_{m}$ represent the volume magnetic susceptibilities of the particle and the medium, respectively. *V* is the volume of the particle, and $B=\mu_{0}H+M$, M is the magnetization and $\mu_{o}$ is the permeability of the vacuum. Another well-known example is inverse ferrofluids [145,146]: these systems can be modeled, in a first approach, as if the magnetic suspension of particles was not present and the non-magnetic particles of radius *R* were instead bearing an induced magnetic dipole $\mu =-4\pi \beta R^{3}H$, where $\beta =\left(\mu_{r}-1\right)/\left(2\mu_{r}+1\right)$ characterizes the effective permeability of the magnetic suspension and $\mu_{r}$ represents the relative permeability of the fluid. This alternative image, used to predict the behavior of inverse ferrofluids, will be very useful when discussing our results although unfortunately Equation (17) cannot be applied to provide a quantitative description in our case. One reason for this limitation is the fact that the particles forming part of the polymeric chains are highly correlated: they are linked forming chains and grafted to a surface, and their density is further perturbed by the non-penetrating particle. Thus, we cannot assume a homogeneous bath of dipolar particles as the use of Equation (17) implies. Another reason is that the aggregates in suspension have a size that cannot be disregarded respective to the size of the non-dipolar particle (the penetrating particle) which would be another condition to be fulfilled for Equation (17) to hold. On the other hand, the grafting surface is not spherical which also further prevents us from using Equation (17).

It should be noted that Kelvin forces are expected to be large in our systems only when an external field is applied. The reason for this is that at zero field we can consider those monomers to exhibit a random orientation of their dipoles: there is always a certain correlation among the orientation of the dipoles due to the fact the chains are grafted to the surface, but it has been shown in previous works that it is very small [118]. Therefore, the polarization/magnetization of the media is almost zero, so Kelvin forces are expected to be almost negligible. When an external field is applied, monomers tend to orientate along the external field, so they form a media with a non-zero polarization/magnetization, and in these cases, Kelvin forces are expected to be of importance to explain the behavior of the systems.

A quite simple qualitative explanation of the features observed in the force profiles when an external field is applied can be drawn if one proceeds by analogy with inverse ferrofluids, and we consider an alternative but equivalent image of our system: forces observed in our system will be similar to those observed in a hypothetical or alternative system in which the volume occupied by the polymer brush is non-dipolar, but both the grafting surface and the penetrating particle are instead made of a dipolar material. In this alternative model, the dipoles inside the dipolar material point roughly in the opposite direction to that of the external field in our real system, and the modulus of those dipoles increases with the α of our real system. Therefore, the surface and penetrating particle have dipoles oriented in a close-to-nose–tail conformation and the force between them will be of an attractive nature. This attractive force will counteract the repulsive one arising from the steric interactions, and competition between these two opposite forces will exist.

Therefore, the existence of a net attractive force for the penetrating particle will happen only when attractive Kelvin forces dominate over the repulsive forces created by the steric hindrance among the monomers of the brush and the penetrating particle. It should be noted that overcoming the attractive forces can only happen when the penetrating particle and the surface are not too far as otherwise Kelvin forces will be too weak. In the same way, attractive forces cannot overcome repulsive ones when the penetrating particle is too close to the surface, since in that case the brush is so locally compressed that the steric hindrance is very high. Additionally, if we increase the grafting density, we then increase the steric hindrance, especially when the particle is close to the grafting surface. We expect that beyond a certain grafting density threshold the repulsive forces will completely dominate over Kelvin forces. In turn, if we reduce the grafting density we certainly reduce the steric hindrance produced by the monomers, but in that case, we also dilute the dipolar media so Kelvin forces also become quite weak in nature. For that reason, our simulations point out that there exists an optimal grafting density that shows the largest net attractive force for the penetrating particle, and it also explains why too low or too high grafting densities are not compatible with having a stationary point in the force profile of the penetrating particle.

**Figure 5:**
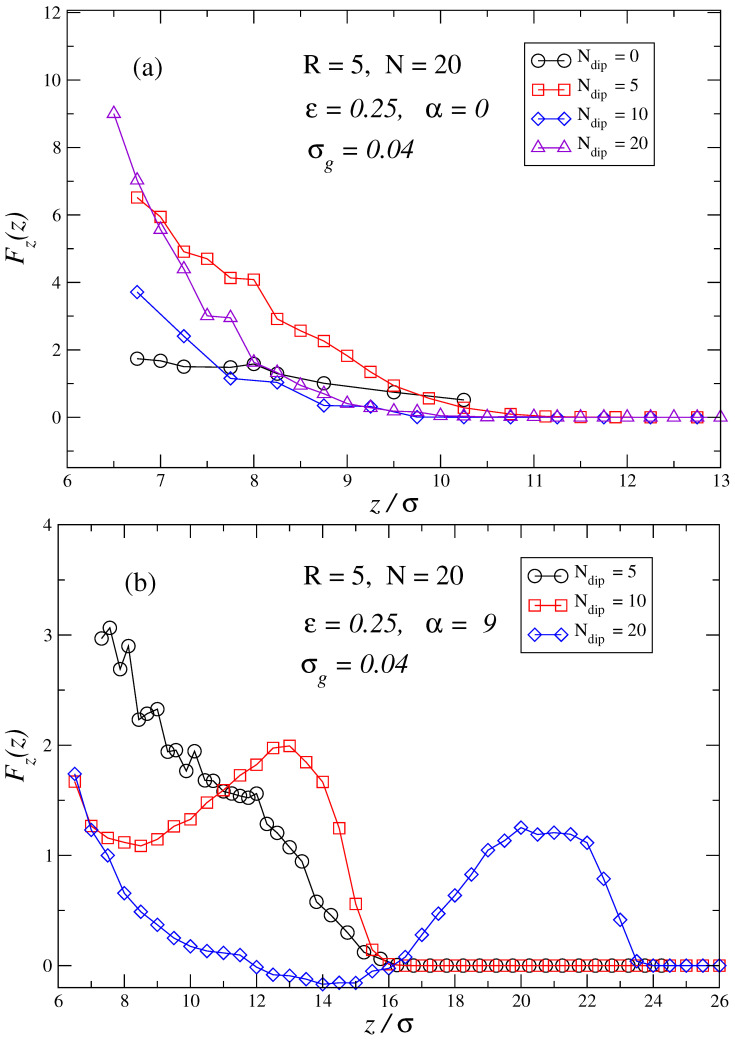
Force profiles representing the force perpendicular to the grafting surface as a function of the distance *z* between the surface and the center of the penetrating particle. Brush parameters are similar to previous figures but now the number of dipoles contained in the chain varies as $N_{dip}=\;0,\;5,\;10,\;20$, corresponding $N_{dip}=20$ to a chain where all monomers bear a dipole. The rest of cases, dipoles are equally spaced inside the monomer sequence of the chain. Plot (**a**) corresponds to (α=9, ε=0), plot (**b**) corresponds to (α=9, ε=0.25).

Another important point to understand is how the dipolar content of the brush at a fixed polymer chain length influences the force profiles felt by the penetrating particle. Figure 5a,b shows the force profiles for brushes with chains of total length N=20 immersed in a bad solvent. The force profiles for brushes made of a different number of dipolar particles in them $N_{dip}=\;0,\;5,\;10,\;20$ are shown. In the zero field case, Figure 5a, the most repulsive force profile for intermediate distances between the particle and the grafting surface corresponds to $N_{dip}=5$. This can be understood as a small amount of sparse dipolar monomers in the chain that helps to increase the persistence length of the chain as segments containing dipoles inside the same chain try to align. Nonetheless, when the amount of dipolar monomers is further increased, the interaction among dipoles of neighboring chains will start to be non-negligible, persistence length will decrease and brushes will start to adopt more collapsed conformations, which leads to having a smaller steric hindrance at the same surface–particle distance. For α=9, as seen in Figure 5b, it is observed that the chains tend to be more extended as $N_{dip}$ increases gradually, developing the typical force profile observed in previous figures. As already explained in the preceding paragraph, a larger dipole content in the brush strengthens the Kelvin forces, which can overcome the repulsive steric force leading to the existence of a net attractive force felt by the penetrating particle. Force profiles for $N=N_{dip}\in \left(5,40\right)$ have also been determined (not shown): these force profiles show similar features to those observed in previous figures but net attractive forces between the penetrating particle and the brush can only be obtained for an intermediate range of polymer lengths. The extension of those ranges depends on the system parameter sets and the strength of the external applied field. This last result can be reasoned as follows: too short chains lead to a dipolar region that is too small to be able to develop Kelvin forces large enough to oppose the repulsive steric forces. Moreover, such short chains do not allow for a proper immersion of the penetrating particle inside the dipolar media, which also produces small Kelvin forces. Inversely, when chains are very long if we resort to the analogy of an inverse system we did before, we find that once the particle is fully immersed in the brush, the surface–particle distance is still too large for Kelvin forces to be strong enough to overcome the steric force. If the particle gets closer to the surface, those Kelvin forces will increase, but the monomer density, and thus the steric force will increase. Therefore, when polymer chains are very large, Kelvin forces will never overcome the steric repulsive forces, so the penetrating particle can never feel a net attractive force.

**Figure 6:**
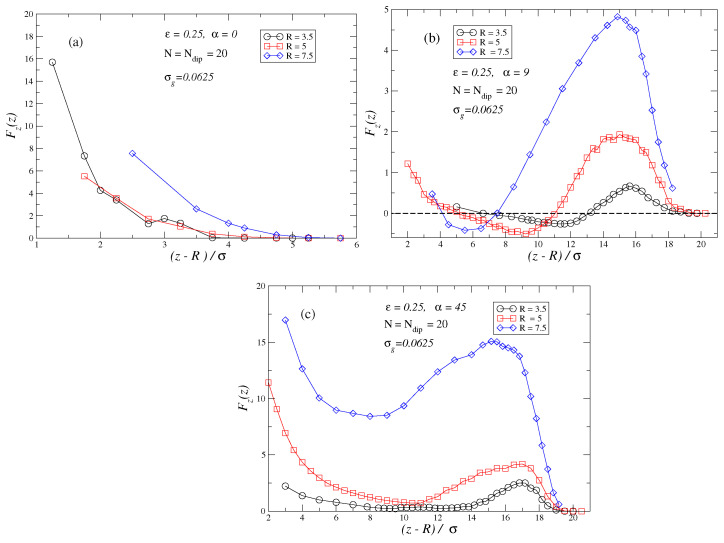
In these plots the dependence of the force profiles as a function of the radius of the penetrating particles is studied for several external fields α=0 in plot (**a**), α=9 in plot (**b**) and α=45 in plot (**c**). Brushes are under bad solvent conditions ϵ=0.25, the grafting density of the brushes is set to $\sigma_{g}=0.0625$ and chains are fully dipolar $N=N_{dip}=20$.

Finally, it is also very interesting to determine how the force profiles depend on the diameter of the penetrating particle. Figure 6a–c shows several field strengths (α=0,9,45), respectively, and how the force profile varies for diameters R=3.5,5,7.5. As expected, we observe that the larger the diameter of the penetrating particle is, the higher the initial repulsive force barrier exerted by the brush on the penetrating particle. Nonetheless, as Figure 6b shows, that does not prevent the existence of a region with attractive forces, although the attractive basin is observed to decrease in range with the particle diameter. The rationale behind that behavior is that although the steric hindrance exerted by the brush increases with the surface of the penetrating particle exposed to the monomers, so does the Kelvin forces which also will increase in strength with the volume of the particle.

## 4. Conclusions

In this work, we have studied, via numerical simulations, the interaction of a penetrating particle with a dipolar colloidal brush. The brush is constructed by grafting many supramolecular polymers onto a common surface, each of which consists of a linked sequence of dipolar and neutral particles (the monomers of the chains). The study has been exemplified using magnetic interactions and magnetic colloidal brushes, but the conclusions of this work can be easily extended to the case of supramolecular and molecular electric dipolar brushes. The dipole–dipole and dipole–external field interactions are the same, except for a prefactor for magnetic and electric dipoles.

Force profiles for penetrating particles of different sizes have been determined for a wide set of grafting densities, solvent conditions, chain lengths and dipolar content of the brushes. In the absence of an external field, dipolar brushes are observed to exhibit rather monotonous force profiles that are a reflection of the steric hindrance created by the compactness of the brushes. As expected bad solvents tend to promote more compactified brush structures, which in turn, produce force profiles of shorter range but with a steep increase in the force as surface–particle distance reduces. An important modification in the force profiles occurs when an external field is applied perpendicular to the grafting surface. In that case, in addition to the expected extension of the chains [118] that translates into an extended action range of the force profiles, a force barrier develops at large distances from the surface. These barriers increase in height with the field, and also with the grafting density: nonetheless, there is a grafting density threshold beyond which the barrier does not grow further in height, rather it decreases slightly. Another important feature of the force profiles under an external field is that there is an intermediate range of surface–particle distances in which repulsion forces are drastically reduced. For bad solvent and moderated field strengths, the forces in this intermediate regime of distances can turn even from repulsive to attractive and lead to the existence of a stationary point in which penetrating particles will tend to remain entrapped inside the brush. This quite unexpected attraction between the brush and the penetrating particle can be reasoned, as explained in further detail in the previous section, as the result of an equilibrium between two opposite forces: the entropic or steric repulsive force due to the high density of monomers near the grafting surface, and an attractive force emerging from Kelvin forces due to the mismatch between the dipolar media created by the dipolar monomers of the brush, and the non-dipolar media existing inside the particle and the grafting surface. Since the strength of both forces depends on the external field applied, it is possible to induce larger or smaller attractive forces onto the penetrating particle by tuning the external field. Some other observed results can also be qualitatively explained through this competition between steric repulsive forces and Kelvin forces: given the grafting density of the chains, there is an optimal value of the external field that maximizes the attraction between the brush and the penetrating particle; brushes with polymer chains that are too short or too long will not lead to regions inside the brush where the brush attracts the penetrating particle; for penetrating particles of a larger size the force barrier becomes increasingly more repulsive, but that fact does not preclude the existence of stable points for the penetrating particle inside the brush.

The possibility to induce force profiles with stationary points via external fields can be used to favor applications of polymer brushes in which the entrapping and retention of colloidal particles for later release is required. Moreover, the possibility to create and control the strength of force barriers at a certain distance from the grafting surface can also be used, among other applications, to control the rate of adsorption and reactivity of catalytic surfaces. The results obtained in this work also envisage the potential use of dipolar brushes in the field of self-assembly for the fabrication of materials based on self-organized colloidal structures, protein crystallization assistance, and shape and size selection of colloidal particles, among other uses.

This study constitutes a first step towards the understanding of the interaction of dipolar colloidal brushes with colloidal particles in a solution. The next steps comprise the study of the force profiles for non-spherical colloids and molecules, as well as the development of analytic frameworks to accurately predict the forces involved and the resulting force profile. We expect our work will stimulate further developments on this subject of increasing scientific interest.

## Data Availability

Data are contained within the article.

## Supplementary Materials

The following supporting information can be downloaded at: https://www.mdpi.com/article/10.3390/polym17030366/s1, Figure S1: Series of instant force measurements for brush–particle interaction for a single run at distances of the grafting surface to the center of the penetrating particle z=8 (top), z=12 (middle), z=16 (bottom). The parameters of the system are: radius penetrating particle R=5, brushes are fully dipolar $N=N_{dip}=20$, Langevin parameter α=9, brush grafting density $\sigma_{g}=\;0.0625$, bad solvent ε=0.25 (sticky chains condition), Temperature T=0.5.
