# Supplementary material for: Dipolar Brush Polymers: A Numerical Study of the Force Exerted onto a Penetrating Colloidal Particle Under an External Field
Source: Polymers (Basel). 2025 Jan 29;17(3):366. doi: 10.3390/polym17030366 (PMC11820698; doi:10.3390/polym17030366)
Supplement: Supplementary file 1 [file polymers-17-00366-s001.zip › polymers-3403471-supplementary.pdf]

Supplementary Materials: Dipolar brush polymers: a numerical study of the force exerted onto a penetrating colloidal particle under an external field

A. Fuster-Aparisi[1], Antonio Cerrato[2], Josep Batle[1], Joan Josep Cerdà[1]* 0000-0003-1132-5989

1. Supplementary material

In this document we show figure S1 that exemplifies the nature of the series of 20000 instant forces collected during the simulation. The three series shown in the plot correspond to three different distances between the grafting surface and the penetrating particle: $z = 8$ (top), $z = 12$ (middle), $z = 16$ (bottom). These series are subsequently averaged, together with the series from another two independent runs, to get the corresponding points in the force profile for the respective values of z. The parameters of the brush-particle system can be found in the caption of the figure S1, and correspond to the case $\sigma_g = 0.0625$ in figure 3b. Positive values of F_z imply that the whole set of polymer chains exert a net total force on the penetrating particle with a component directed outwards the brush (repulsive force). If the force has a negative value the sum of all the forces exerted by the polymer chains on the penetrating particle has a component towards the grafting surface of the brush (attractive force).

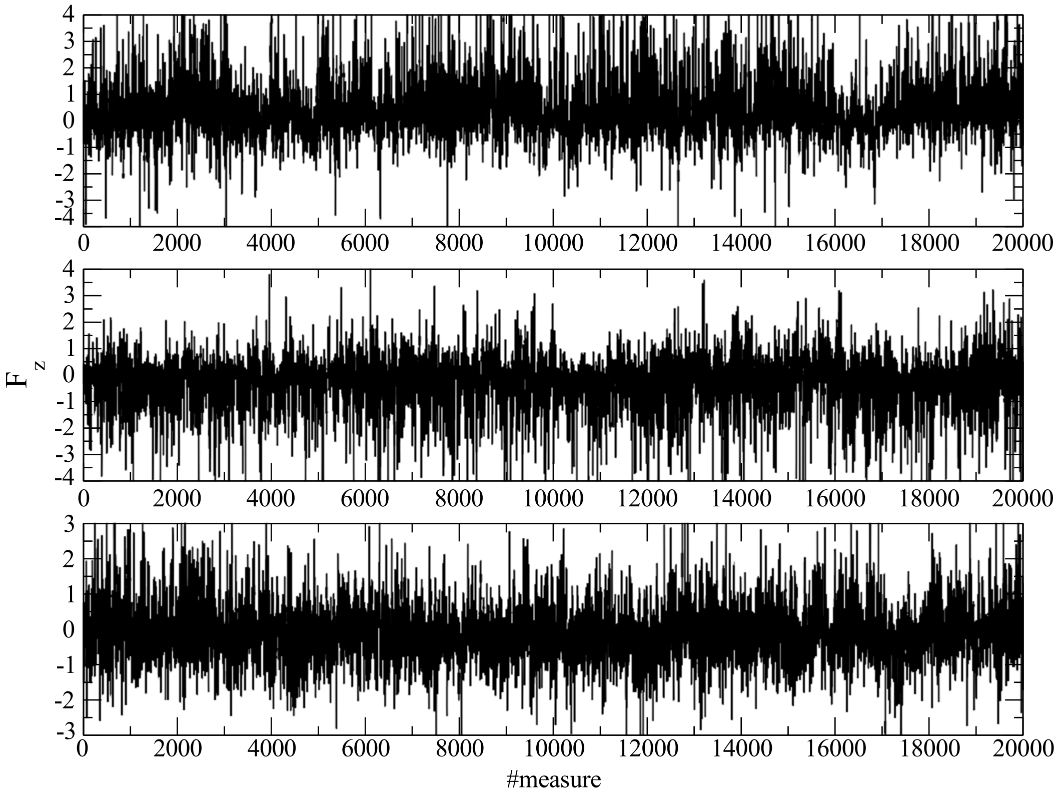

Figure S1. Series of instant force measurements for brush-particle interaction for a single run at distances of the grafting surface to the centre of the penetrating particle $z = 8$ (top), $z = 12$ (middle), $z = 16$ (bottom). The parameters of the system are: radius penetrating particle $R = 5$, brushes are fully dipolar $N = N_{dip} = 20$, Langevin parameter $\alpha = 9$, brush grafting density $\sigma_g = 0.0625$, bad solvent $\varepsilon = 0.25$ (sticky chains condition), Temperature $T = 0.5$.

Author Contributions: Conceptualization, J.C.; methodology, J.C. .; software, J.C., A.F.; validation, A.C. and J.B.; formal analysis, J.C., A.F. ; investigation, J.C., A.F., A.C., J.B; resources, J.C ; data curation, J.C. ; writing—original draft preparation, A.F. and J.C.; writing—review and editing, A.C, J.B. ; visualization, J.C.; supervision, J.C.; project administration, J.C. All authors have read and agreed to the published version of the manuscript.

Funding: All authors thank the financial support of the Spanish Ministry of Economy and Competitiveness (MINECO/AEI/FEDER,UE) through the projects *Proyecto de I+D (excelencia) DPI2017-86610-P* and *PID2020-118317GB-I00 MICIU/AEI /10.13039/501100011033*.

Institutional Review Board Statement: Not applicable.

Informed Consent Statement: Not applicable.

Data Availability Statement: Dataset available on request from the authors.

Acknowledgments: All authors thank Pedro Sánchez from the University of Wien for his careful reading of the manuscript.

Conflicts of Interest: The authors declare no conflicts of interest.

Disclaimer/Publisher's Note: The statements, opinions and data contained in all publications are solely those of the individual author(s) and contributor(s) and not of MDPI and/or the editor(s). MDPI and/or the editor(s) disclaim responsibility for any injury to people or property resulting from any ideas, methods, instructions or products referred to in the content.
